# Modulating Mesenchymal Stem Cell Behavior Using Human Hair Keratin-Coated Surfaces

**DOI:** 10.1155/2015/752424

**Published:** 2015-06-01

**Authors:** Pietradewi Hartrianti, Ling Ling, Lyn Mei Ming Goh, Kok Seng Amos Ow, Rebekah Margaret Samsonraj, Wan Ting Sow, Shuai Wang, Victor Nurcombe, Simon M. Cool, Kee Woei Ng

**Affiliations:** ^1^School of Materials Science and Engineering, Nanyang Technological University, Singapore 639798; ^2^Institute of Medical Biology, Agency for Science Technology and Research (A^*^STAR), Singapore 138648; ^3^School of Biological Sciences, Nanyang Technological University, Singapore 637551; ^4^Department of Orthopaedic Surgery, National University of Singapore, Singapore 119288

## Abstract

Human mesenchymal stem cells (hMSCs) have shown great potential for therapeutic purposes. However, the low frequencies of hMSCs in the body and difficulties in expanding their numbers *in vitro* have limited their clinical use. In order to develop an alternative strategy for the expansion of hMSCs *in vitro*, we coated tissue culture polystyrene with keratins extracted from human hair and studied the behavior of cells from 2 donors on these surfaces. The coating resulted in a homogeneous distribution of nanosized keratin globules possessing significant hydrophilicity. Results from cell attachment assays demonstrated that keratin-coated surfaces were able to moderate donor-to-donor variability when compared with noncoated tissue culture polystyrene. STRO-1 expression was either sustained or enhanced on hMSCs cultured on keratin-coated surfaces. This translated into significant increases in the colony-forming efficiencies of both hMSC populations, when the cells were serially passaged. Human hair keratins are abundant and might constitute a feasible replacement for other biomaterials that are of animal origin. In addition, our results suggest that hair keratins may be effective in moderating the microenvironment sufficiently to enrich hMSCs with high colony-forming efficiency *ex vivo*, for clinical applications.

## 1. Introduction

Multipotent, self-renewing human mesenchymal stem cells (hMSCs) are promising tools for tissue engineering and regenerative medicine because of their capability to differentiate into numerous tissue lineages, including bone, cartilage, fat, and fibrous connective tissue [1]. They have been obtained from multiple mature tissues, including bone marrow, umbilical cord, and adipose tissue [2], and raise fewer ethical issues than embryonic stem cells. Despite such promise, there are few hMSC clinical applications that have obtained regulatory approval. Obstacles for their widespread use include limited numbers of autologous hMSCs that can be harvested from any given tissue and the loss of multipotency during* in vitro* expansion [3, 4]. There is thus a pressing need for strategies that enable the expansion of hMSC numbers* in vitro* without compromising their self-renewal and differentiation capabilities.

Significant efforts have been put into developing substrates that support greater levels of hMSC attachment, proliferation, and maintenance of multipotency. Materials such as poly-L-lysine, fibronectin, laminin, and collagen can, when coated onto culture surfaces, all support effective maintenance of hMSCs* in vitro* [5–7]. Of these, fibronectin coating is one of the most widely used. The glycoprotein fibronectin is a major component of many extracellular matrices, within which it is responsible for supporting cell adhesion. It contains binding sequences for both proteoglycans and the integrins *α*_*5*_ *β*_*1*_, *α*_*IIb*_ *β*_*3*_, *α*_*IIb*_ *β*_*1*_, *α*_*IIb*_ *β*_*3*_, and *α*_*4*_ *β*_*1*_ [8]. Laminin, an abundant glycoprotein within basal laminae, has many bioactivities and has been shown to enrich osteoblast progenitors in fetal rat calvaria cells* in vitro* [9, 10]. Collagen I-coated surfaces have been demonstrated to increase MSC proliferation [11].

Similarly to fibronectin, human hair keratins contain the LDV (Leu-Asp-Val) cell adhesion motif recognized by integrin *α*_*4*_ *β*_*1*_ [12, 13]. Keratins belong to the family of intermediate filament proteins found in wool, nails, hooves, horns, feathers, and human hair [14]. Although they are thought to serve mainly structural purposes, they are now known to be implicated in mechanotransduction pathways and their mutations are the cause of several epithelial diseases in humans [15]. They possess distinct advantages as biomaterials, including abundance, biodegradability, intrinsic bioactivity, and the fact that they are a viable source of autologous human material that can be harvested [16]. In recent years, there has been an increasing interest in the application of human hair keratins for such biomedical purposes as wound dressing, tissue engineering, and drug delivery. Although keratins have been fabricated into a variety of formats, including hydrogels, fibers, and films [14, 17, 18], little has been reported about their potential as a surface coating to enhance cellular behavior. We have previously shown that keratin-coated surfaces encourage mouse fibroblasts to express greater amounts of fibronectin on tissue culture polystyrene, suggesting that they could act as extracellular stimuli to evoke specific cell responses [19]. Here we evaluated the influence of keratin-coated surfaces on hMSC function. Our results indicate that keratins can indeed modulate hMSC activity and deserve further optimization to develop their capabilities.

## 2. Materials and Methods

### 2.1. Extraction of Human Hair Keratin

Discarded human hair was obtained from local salons in Singapore, mixed, washed extensively with detergent, and further rinsed with ethanol before air-drying at room temperature. The dried hair was then delipidized by soaking in a mixture of chloroform and methanol (2 : 1, v/v) for 24 h. Subsequent keratin extraction was done by mixing the hair into 0.125 M of sodium sulfide (Na_2_S; Sigma-Aldrich) in deionized water and incubating the mixture for 4 h at 40°C. The resulting mixture was then filtered and dialyzed against 5 L of deionized water in cellulose tubing (MWCO: 12400; Sigma-Aldrich). Protein concentration of the extract was measured with the 660 nm protein assay (Thermo Scientific, USA) according to the manufacturer's instructions.

### 2.2. Sodium Dodecyl Sulfate Polyacrylamide Gel Electrophoresis (SDS-PAGE) and Coomassie Blue Staining

To determine sample quality by SDS-PAGE, 20 *μ*g of the extracted keratin samples was mixed with 5 *μ*L of lithium dodecyl sulfate (LDS) sample buffer (Invitrogen, USA) and 2 *μ*L of sample reducing agent (DTT, 10X) (Invitrogen, USA) and topped up to a total volume of 20 *μ*L with deionized water. Each sample was denatured by heating at 75°C for 10 min before loading into precast NuPAGE 4–12% Bis-Tris Gels (Invitrogen, USA). Electrophoresis was carried out at a constant voltage of 120 V for 90 min in 0.05% NuPAGE morpholinepropanesulfonic acid (MOPS) SDS running buffer (Invitrogen, USA). Subsequently, separated proteins in the gel were stained with SimplyBlue Coomassie SafeStain (Invitrogen, USA) for 60 min and destained in deionized water on a shaker overnight. Prior to visualization, the gel was dried with DryEase Mini-Gel Drying System (Invitrogen, USA).

### 2.3. Keratin Coating

Nunclon Δ surface tissue culture polystyrene (TCPS) dishes (100 mm; Thermo Scientific, USA) and 24-well Nunclon Δ surface TCPS plates (Thermo Scientific, USA) were coated with 80 *μ*g/mL (4 mL and 138 *μ*L, resp.) of keratin solution overnight at 4°C. Following coating, the surfaces were washed once with phosphate buffered saline (PBS) and left to stand for 15 min at room temperature. Uncoated dishes and wells were used as negative controls.

### 2.4. Water Contact Angle Measurement

Static water contact angle was measured at room temperature, for both keratin-coated and uncoated (control) surfaces, using the FTA32 Contact Angle and Surface Tension Analyzer (Analytical Technologies, Singapore). A total of twelve measurements for each surface were recorded.

### 2.5. Atomic Force Microscopy (AFM)

Surface topography was recorded using single-beam silicon cantilever probes (Veeco RTESP: resonance frequency 300 KHz, nominal tip radius of curvature 10 nm, and force constant 40 N/m) in tapping mode, on the Nanoscope IIIa (Veeco Instruments, USA) atomic force microscope.

Mean surface roughness values (*R*_*a*_) of four random fields per sample, from 3 independent experiments, were calculated using Nanoscope 6.13R1 software (Digital Instruments, USA). Where necessary, data sets were subjected to first-order flattening before recording *R*_*a*_ values.

### 2.6. Human Mesenchymal Stem Cell (hMSC) Culture

Human MSCs were isolated, as described previously [20] from human bone marrow mononuclear cells (BMMNCs; Lonza, USA) from two young male donors of age range 20–23, referred herein as Donors A and B. Human MSCs isolated were characterized [21] before use. The cells were maintained in hMSC maintenance media, which is made up of low glucose (1000 mg/L) Dulbecco's Modified Eagle Media (DMEM; Biopolis Shared Facilities, A∗STAR, Singapore), 10% fetal calf serum (FCS) (Hyclone, USA), 100 units/mL penicillin-streptomycin (Gibco, Invitrogen, USA), and 2 mM L-glutamine (Gibco, Invitrogen, USA), at 37°C and 5% CO_2_. Cells were seeded at 3,000/cm^2^ at every passage and cultured over 2 or 3 passages on keratin-coated and uncoated dishes until they reached subconfluency, before being subjected to attachment, proliferation, and marker expression assessments.

### 2.7. Cell Attachment Assay

Cells were seeded at 3,000 per cm^2^ on plates coated with or without 80 *μ*g/mL keratin and allowed to adhere for 2 or 6 h. Thereafter the unattached cells were removed by PBS and the remaining attached cells were lifted using 0.125% trypsin-ethylenediaminetetraacetic acid (EDTA) and counted using the GUAVA ViaCount FLEX reagent and ViaCount Assay Software on the Guava easyCyte 8HT Benchtop Flow Cytometer (Merck Millipore, USA), as indicated in the supplier's instructions.

### 2.8. Cell Proliferation Assay

Serially passaged human MSCs (passages 5 to 7) were removed from the wells using 0.125% trypsin-EDTA upon subconfluency. Cell viability and numbers were then determined by the GUAVA ViaCount system as described above.

### 2.9. Flow Cytometry

To determine hMSC phenotype, CD49a and STRO-1 levels were evaluated by flow cytometry. Human MSCs at passage 5 or 7 were removed from the dishes using TrpLE Select (Gibco, Invitrogen, USA) and neutralized with hMSC maintenance media. Subsequently, they were washed and resuspended in the respective primary antibody diluted in staining buffer, which consists of 2% FCS and 0.01% (w/v) sodium azide (Sigma-Aldrich, USA) in PBS. Phycoerythrin- (PE-) conjugated mouse anti-human CD49a primary antibody (BD Biosciences, USA) was diluted in the staining buffer at 1 : 50 and incubated with the hMSCs for 60 min at 4°C in the dark. Cells were then washed twice before being resuspended in staining buffer and analyzed with a BD FACSArray Bioanalyzer. The STRO-1 primary antibody was kindly provided by Professor Stan Gronthos, School of Medical Sciences, Faculty of Health Sciences, University of Adelaide, Australia, as hybridoma supernatant and incubated directly without dilution on the hMSCs for 45 min at room temperature. Cells were then probed with PE-conjugated goat anti-mouse IgM (Invitrogen, USA) for 45 min at 4°C in the dark, before being washed and analyzed as described above. Data obtained was analyzed with FlowJo Software version 7.6.5 (Tree Star, Inc., USA), at 2% gating of the respective isotype controls. The relevant isotypes of the primary antibodies were used as negative controls. Experiments were performed in triplicates and results represented as the mean percentage of cells positive for the surface markers analyzed.

### 2.10. Colony-Forming Unit Fibroblast (CFU-F) Assay

For the CFU-F assay, 150 hMSCs at passage 5 or 7 were seeded per 100 mm dish and maintained over a growth period of 14 days. Media were changed on the 7th day and every 2 days thereafter. Cell culture was terminated on the 14th day and colonies were stained using 0.5% (w/v) crystal violet (Sigma-Aldrich, USA) in 100% methanol (Merck, USA) and sequentially rinsed with PBS and deionized water. Colonies on each dish were counted by two different individuals blinded to the sample identities. Only colonies that were not in contact with neighboring colonies and comprised of more than 50 cells or with a diameter of ≥2 mm were taken into account.

### 2.11. Statistical Analysis

All quantitative values were expressed as means ± standard deviation, with *n* = 3 or 6, depending on the experiments. Statistical analyses were performed using Student's *t*-test or one-way ANOVA, with the aid of GraphPad Prism version 6 (GraphPad Software, USA). *P* values less than 0.05 indicate significant differences.

## 3. Results and Discussion

The limited availability of hMSCs remains a significant stumbling block on the path to realizing their full clinical potential. The use of advanced cell culture strategies, in combination with the new generation of biomaterials, is clearly the way forward so that adequate stem cell numbers can be obtained in the shortest time possible [4, 22]. Although coating surfaces with animal-derived ECM proteins has a long provenance in the literature, such approaches will hinder their eventual translation due to regulatory restrictions. Thus we sought here to examine a novel approach, that of coating TCPS using keratins derived from human hair.

### 3.1. Fabrication and Characterization of Keratin-Coated Tissue Culture Polystyrene

**Figure 1:**
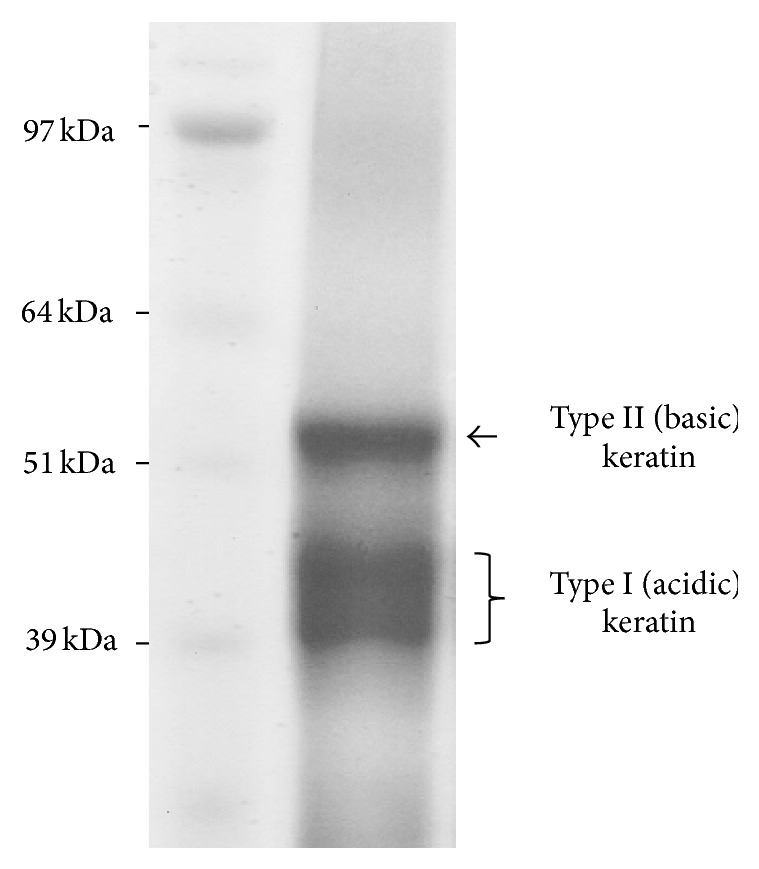
SDS-PAGE analysis of proteins extracted from hair.* Lane 1*: protein ladder;* Lane 2*: representative coomassie blue-stained sample of hair-extracted proteins, where the presence of two dominant fractions of keratins is evident.

The keratin extraction protocol exploited here yielded a solution concentration of ~20 mg/mL. Consistent with previous work, coomassie blue-stained SDS-PAGE gels revealed the presence of two distinct fractions within the extracted samples (Figure 1). Using Western Blotting, we have previously demonstrated that the two bands between 39 and 45 kDa are type I (acidic) keratins, while the single band at 50–55 kDa is type II (basic) keratin [17, 23].

**Figure 2:**
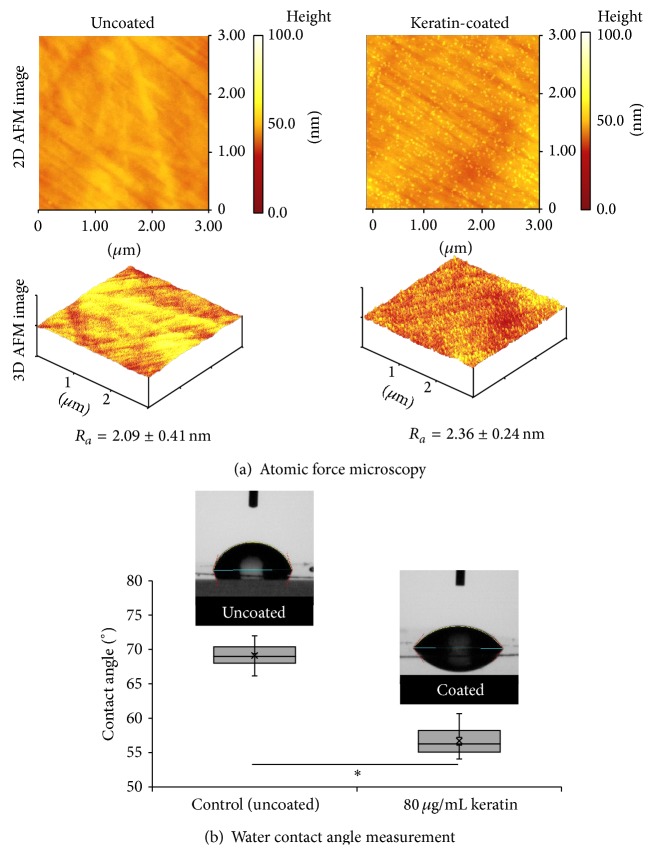
Surface characteristic of uncoated and keratin-coated TCPS. (a) 2D and 3D AFM images showing topography of surfaces and mean roughness (*R*_*a*_). (b) Water contact angle measurement showing increased hydrophilicity upon keratin coating; ^∗^ *P* < 0.05.

The surface topography of the uncoated and keratin-coated TCPS was studied using AFM. As shown in Figure 2(a), the uncoated TCPS surface exhibited a series of randomly oriented line features which were artefacts on the original culture plate material. These were also observed in the background of the keratin-coated surfaces. However, in addition to these, the keratin-coated surfaces displayed a layer of random, but evenly distributed, punctate features with diameters of 40–50 nm. These features were nanosized keratin globules that were clearly observed in the 3D AFM images as well. The mean roughness (*R*_*a*_) of the uncoated and keratin-coated TCPS surfaces was 2.09 ± 0.41 nm and 2.36 ± 0.24 nm, respectively. We had previously showed that nanosized keratin globules on cell culture surfaces enhanced fibronectin production in fibroblasts [19]. With MSCs, McMurray et al. showed that nanoscale features processed into polycaprolactone by electron beam lithography facilitated their growth [24]. Similarly, human embryonic stem cells would also respond to differing surface nanotopography [25]. A measurement of water contact angle was performed to confirm the effect of keratin adsorption on surface hydrophilicity. As shown in Figure 2(b), the mean water contact angles were 69.1 ± 1.9° for uncoated TCPS and 56.7 ± 2.1° for keratin-coated TCPS, suggesting that a more hydrophilic surface is created after keratin adsorption.

### 3.2. Cell Attachment and Proliferation

**Figure 3:**
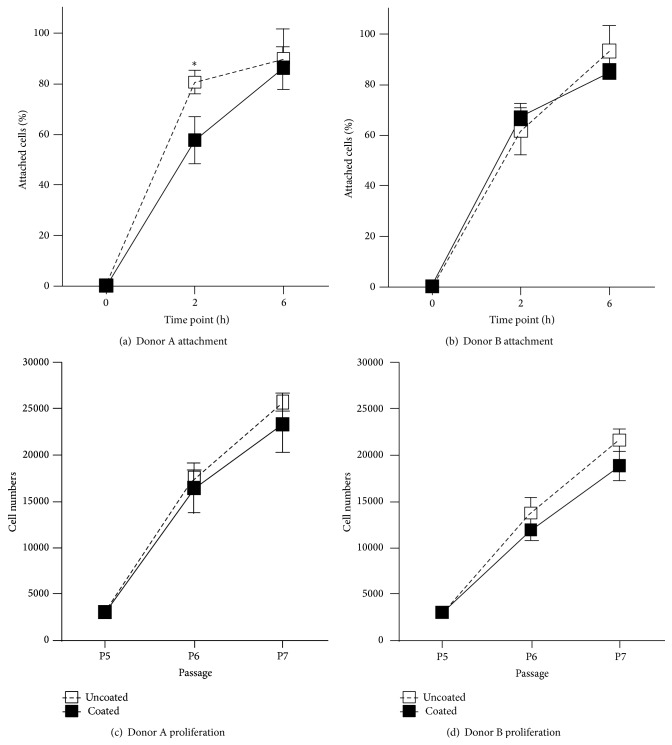
Cell attachment and proliferation. Percentage of attachment of hMSCs from (a) Donor A and (b) Donor B on uncoated and keratin-coated TCPS surfaces over 2 and 6 h; *n* = 3, ^∗^ *P* < 0.05. Cumulative cell numbers of hMSCs from (c) Donor A and (d) Donor B on uncoated and keratin-coated TCPS surfaces over 2 passages; *n* = 6.

The two populations of hMSCs used in this study came from individuals of the same gender and age group. Despite this, differences in cell response were observed, presumably due to inherent genetic variation. On uncoated surfaces, donor-to-donor variability in cell attachment efficiency was clearly evident (Figures 3(a) and 3(b)). Two hours after seeding, ~80% of the hMSCs from Donor A had already attached onto the uncoated surfaces, compared to only ~62% of Donor B's hMSCs. However, this difference became insignificant after 6 hours, where both sets of hMSCs registered close to 90% attachment. In contrast, keratin-coated surfaces appeared to have mitigated this donor variability. Both sets of hMSCs showed similar attachment rates, reaching ~60% after 2 hours and ~80% after 6 hours. Both hMSC populations recorded comparable proliferation rates over three passages (P5 to P7) on uncoated and keratin-coated surfaces (Figures 3(c) and 3(d)). This suggests that keratin did not compromise the proliferative capacities of cells from either donor.

### 3.3. Expression of Stem Cell Markers

**Figure 4:**
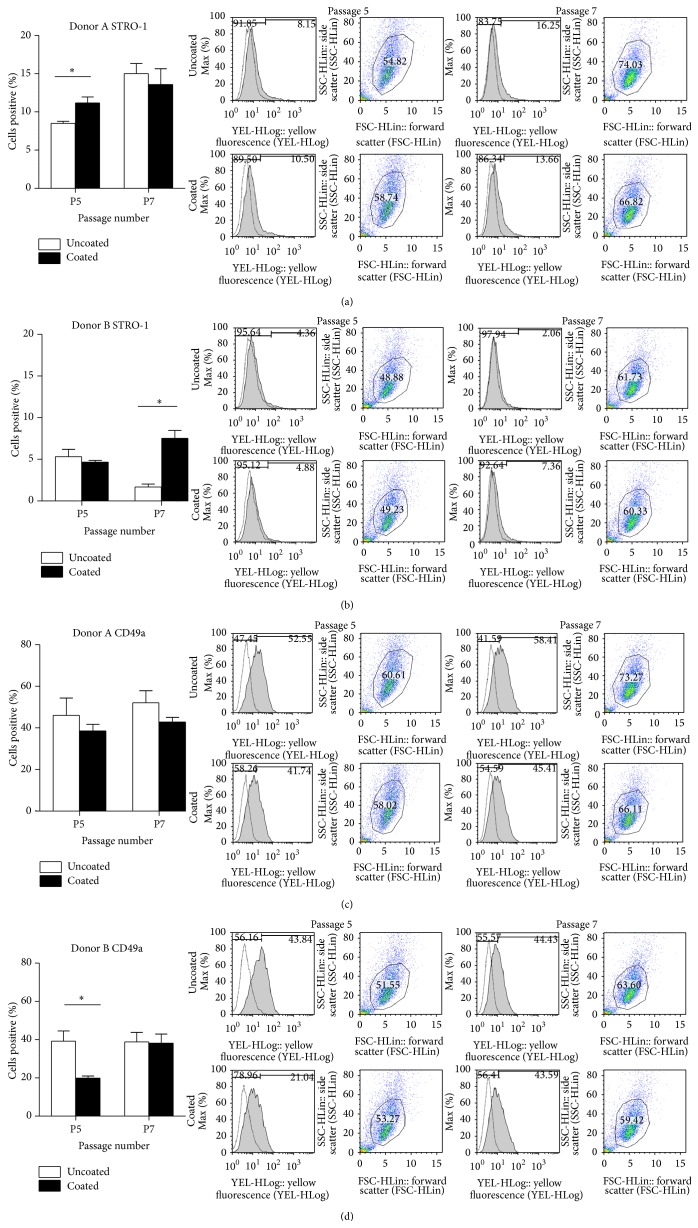
Flow cytometry analysis of stem cell marker expression. Human MSCs from Donor A and Donor B were cultured on uncoated or keratin-coated surfaces from passage 4 to passage 7. The stem cell markers STRO-1 (a and b) and CD49a (c and d) were assessed at passage 5 and passage 7. All plots show relative percentage expression levels. Representative scatter plots (FSC, *x*-axis and SSC, *y*-axis) show gating of live cells, while histograms show the distribution of expression of the surface markers; *n* = 3, ^∗^ *P* < 0.05.

STRO-1 and CD49a were chosen as the key hMSC biomarkers in this study; CD49a, the *α*_1_-integrin subunit, regulates MSC adhesion to ECM proteins such as collagen and laminin [21, 26, 27]. Here, the initial levels of STRO-1 expression varied between 5 and 8% for both MSC populations (Figure 4(a)), which was in the expected range for viable primary cells [28, 29]. STRO-1 expression in Donor A's hMSCs at passage 5 was significantly higher on keratin-coated surfaces compared to uncoated surfaces. No differences were observed in STRO-1 expression at passage 7 in Donor A cells. For Donor B, STRO-1 expression improved by 3% when cultured on the keratin-coated surfaces over two passages (P5 to P7), whereas expansion on uncoated surfaces showed a steady decline (Figure 4(b)). Similarly to the cell attachment results, keratin-coated surfaces mitigated donor-to-donor variability and could at minimum sustain and sometimes enhance STRO-1 expression.

CD49a expression in Donor A cultures on keratin-coated surfaces appeared to be lower than on uncoated surfaces at both passages 5 and 7 (Figure 4(c)), although this difference was not statistically significant. In comparison, expression of CD49a for Donor B on keratin-coated surfaces was half of that on uncoated surfaces at passage 5 (Figure 4(d)). However, by passage 7 the expression levels of CD49a on keratin-coated surfaces had increased from 19% to 38%, which was comparable to levels that have been previously reported [21] and was similar to the expression levels on uncoated surfaces. Considered collectively, it seemed that while CD49a expression was somewhat suppressed, hMSC adhesion and proliferation were not compromised when cultured on keratin-coated surfaces (Figures 3(a) and 3(b)). This suggests that hMSC adhesion to keratin-coated surfaces was being mitigated through other integrin *α*-subunits, perhaps the *α*_*4*_ variant, which is also known to recognize the LDV cell adhesion motif present in hair keratins [13].

### 3.4. Colony-Forming Efficiency

**Figure 5:**
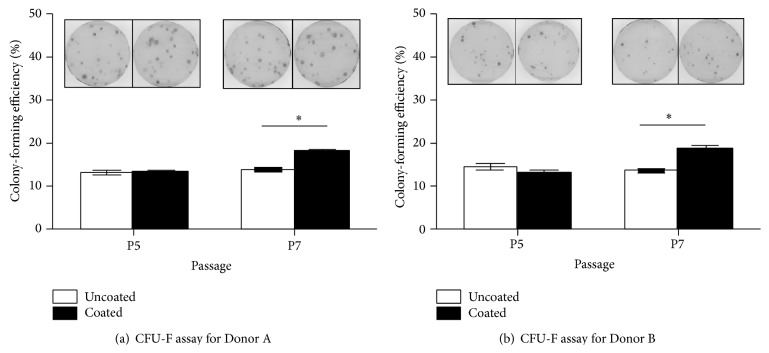
Colony-forming efficiencies of (a) Donor A and (b) Donor B hMSCs after being expanded on either uncoated or keratin-coated TCPS for 1 passage (P4 to P5, assessed at P5) and 3 passages (P4 to P7, assessed at P7). Corresponding micrographs above the bar charts depict representative hMSC colonies stained with crystal violet; *n* = 6, ^∗^ *P* < 0.05.

STRO-1 expression is not only associated with the identification [30] and maintenance of hMSC stemness [26, 31], but also implicated in tissue-specific paracrine signalling. Psaltis et al. recently demonstrated that STRO-1-positive hMSCs release greater levels of cytokines which were capable of increasing cardiac cell proliferation and tube formation by endothelial cells [32]. It is also known that expression of STRO-1 is strongly correlated with the colony-forming ability of hMSCs [31]. Indeed, our CFU-F assay results showed that the two donor hMSCs with sustained or increased STRO-1 expression levels, when cultured on keratin-coated surfaces, also formed more colonies over the same passages. As shown in Figure 5, the efficiencies of colony formation on all cultures showed no significant difference after just one passage on different surfaces (passage 5). However, by passage 7, after being expanded for 3 consecutive passages on the different surfaces, the colony-forming efficiencies of hMSCs from both donors cultured on keratin-coated surfaces increased by ~25%, while those on uncoated surfaces remained unchanged.

Many strategies have been utilized in attempts to maintain stem cell quality during* in vitro* expansion [22], with reports from the literature about biomaterial-coated surfaces being somewhat variable and sometimes contradictory. Biomaterials that have shown promise include collagen, laminin, fibronectin, and a variety of heterogeneous decellularized matrices [5–11]. A confounding factor is the nature of the underlying substrate on which the coating is being laid. The surface and mechanical properties of this substrate will need to be considered carefully so as to provide fair comparisons between different coating materials. Song et al. demonstrated that human collagen type-1-coated silicone membrane surfaces resulted in decreased hMSC proliferation. In contrast, human fibronectin coated on the same silicone membrane surfaces enhanced hMSC attachment but did not influence their proliferation [33]. Here, keratin coating was performed over rigid TCPS surfaces. In a similar experiment, Qian and Saltzman found that TCPS surfaces coated with either collagen, laminin, or fibronectin did not result in any difference in terms of expansion and neuronal differentiation of MSCs. In comparison, TCPS surfaces coated with 50 mg/cm^2^ of Matrigel, which primarily consists of murine laminin, collagen type IV, and heparan sulfate proteoglycans, not only enhanced neuronal differentiation but also significantly improved the proliferative capacity of MSCs [5]. Here, coating TCPS with hair keratin solutions at 80 *μ*g/mL resulted in a significantly lower coating density of 650 ng/cm^2^ [19], which led to effective maintenance of STRO-1 expression with an increased colony-forming efficiency.

## 4. Conclusion

Mesenchymal stem cells from two human donors were cultured on 2D TCPS surfaces coated with human hair keratins at 80 *μ*g/mL. Coated surfaces increased surface roughness and increased hydrophilicity; they were also able to moderate donor-to-donor variability in terms of cell attachment efficiencies while not compromising their proliferative capacities. Human MSC attachment onto keratin-coated surfaces was not regulated through CD49a. Most importantly, STRO-1 expression in both hMSC populations was either maintained or significantly increased on keratin-coated surfaces, which translated into higher colony-forming efficiencies. Together, these results demonstrate for the first time the potential of using keratin-coated surfaces to enrich naïve and functional hMSCs.
